# Impact of Comorbidities on Mortality in Patients with Idiopathic Pulmonary Fibrosis

**DOI:** 10.1371/journal.pone.0151425

**Published:** 2016-03-29

**Authors:** Michael Kreuter, Svenja Ehlers-Tenenbaum, Karin Palmowski, Jacques Bruhwyler, Ute Oltmanns, Thomas Muley, Claus Peter Heussel, Arne Warth, Martin Kolb, Felix J. F. Herth

**Affiliations:** 1 Centre for Interstitial and Rare Lung Diseases, Pneumology and Respiratory Critical Care Medicine, Thoraxklinik, University of Heidelberg, Heidelberg, Germany; 2 ECSOR Department of Biostatistics, Gembloux, Belgium; 3 Department of Translational Research, Thoraxklinik, University of Heidelberg, Heidelberg, Germany; 4 Diagnostic and Interventional Radiology, Thoraxklinik, University of Heidelberg, Heidelberg, Germany; 5 Department of Radiology, University of Heidelberg, Heidelberg, Germany; 6 Institute of Pathology, University of Heidelberg, Heidelberg, Germany; 7 McMaster University, Hamilton, ON, Canada; 8 Member of the German Center for Lung Research, Translational Lung Research Centre, Heidelberg, Germany; University of North Dakota, UNITED STATES

## Abstract

**Introduction:**

Comorbidities significantly influence the clinical course of idiopathic pulmonary fibrosis (IPF). However, their prognostic impact is not fully understood. We therefore aimed to determine the impact of comorbidities, as individual and as whole, on survival in IPF.

**Methods:**

The database of a tertiary referral centre for interstitial lung diseases was reviewed for comorbidities, their treatments, their frequency and survival in IPF patients.

**Results:**

272 patients were identified of which 12% had no, 58% 1–3 and 30% 4–7 comorbidities, mainly cardiovascular, pulmonary and oncologic comorbidities. Median survival according to the frequency of comorbidities differed significantly with 66 months for patients without comorbidities, 48 months when 1–3 comorbidities were reported and 35 months when 4–7 comorbidities were prevalent (p = 0.004). A multivariate Cox proportional hazard analyses identified other cardiac diseases and lung cancer as significant predictors of death, gastro-oesophageal reflux disease (GERD) and diastolic dysfunction had a significant positive impact on survival. A significant impact of comorbidities associated therapies on survival was not discovered. This included the use of proton pump inhibitors at baseline, which was not associated with a survival benefit (p = 0.718). We also established a predictive tool for highly prevalent comorbidities, termed IPF comorbidome which demonstrates a new relationship of IPF and comorbidities.

**Conclusion:**

Comorbidities are frequent in IPF patients. Some comorbidities, especially lung cancer, mainly influence survival in IPF, while others such as GERD may inherit a more favourable effect. Moreover, their cumulative incidence impacts survival.

## Introduction

Idiopathic pulmonary fibrosis (IPF) is a chronic, progressive and irreversible lung disease of unknown aetiology [1]. It is the most common form of idiopathic interstitial lung disease, with a prevalence of 14 to 63 per 100.000 (reviewed in [2]), mainly diagnosed in the elderly where the prevalence increases dramatically and a predominance in men and ex/current smokers [1, 3]. The disease is characterised by progressive worsening of lung function, significantly affects health-related quality of life and carries a prognosis that is worse than that of many cancers [4]. The five-year survival rate lies between 20% and 40% and the median survival time is between two and five years [4].

IPF has been associated with a large number of comorbidities such as pulmonary hypertension [5–7], emphysema [8, 9], lung cancer [10, 11], coronary artery disease (CAD) [12, 13], diastolic dysfunction [14], gastroesophageal reflux disease (GERD) [15, 16], sleep disorders [17–19], endocrine disorders and psychiatric disturbances [20]. Some of these conditions can develop as consequence of IPF (e.g. pulmonary hypertension), and others may be explained by common risk factors (e.g. smoking as a potential cause of lung cancer in IPF or the combined pulmonary fibrosis and emphysema). Others, particularly GERD are even discussed as potential cause for IPF, especially the episodes of acute exacerbation. In contrast, others such as sleep apnoea or depression are more difficult to explain. Regardless of the underlying cause, symptoms and quality of life in IPF patients can be significantly influenced by these comorbidities, especially in IPF patients suffering from multiple of these conditions. Some of the studies reported a significant association between comorbidities and survival in IPF [5, 6, 8, 9, 11, 12, 15, 16].

Death in IPF is most often attributed to IPF directly or to pulmonary complications, such as pneumonias, but can also occur due to comorbidities. Major non-respiratory reasons for deaths are related to cardiac disease (heart failure or coronary artery disease), stroke and cancer [4].

The current ATS/ERS/JRS/ALAT guideline for diagnosis and management of IPF states, that patients with IPF may have sub-clinical or overt comorbid conditions such as pulmonary hypertension, gastroesophageal reflux, obstructive sleep apnoea, obesity, and emphysema. However, the impact of these conditions on the outcome of patients with IPF is still uncertain [1, 21].

Aim of our analysis was therefore to determine whether the number of comorbidities, and which specific comorbidities when considered together, impact survival in IPF.

## Methods

### Patients

The database of our tertiary referral centre for interstitial lung diseases (ILD) was reviewed for comorbidities, their frequency and impact on survival in IPF patients. The study included patients diagnosed between 1/2004 until 4/2012.

Comorbidities and their treatments were assessed at baseline visit at our centre and were systematically recorded through direct questioning of the patients at baseline visit supported by a standard questionnaire for lung diseases which includes all respiratory diseases and all organ systems systematically [22]. The patients’ reports on comorbidities were confirmed by reviewing the patient’s medication list, medical reports of the general practitioner and of other physicians / hospitals or when indicated by confirmatory tests. Assessment of patients also included PH was actively screened for by echocardiography and BNP. The further retrospective assessments incorporated baseline demographics, including age, gender, smoking habits (pack years), familial ILD history (also by questionnaire [22]), pulmonary function tests (absolute and % predicted, Vital Capacity [VC], forced expiratory volume in 1 second [FEV_1_], FEV_1_/VC ratio and DL_CO_) and diagnostic procedures (HRCT with patterns [23] and surgical lung biopsy with patterns). The results of all examinations were discussed in a multidisciplinary board consisting of clinical, radiological and pathological experts in the diagnosis of ILDs. The ethics committee of the University of Heidelberg approved this retrospective study. Due to the retrospective nature of this analysis and according to the vote of the ethics committee, written informed consent could not be obtained by the patients but patient records / information were anonymized and de-identified prior to analysis.

### Survival

The follow-up time for each subject was determined from the date of baseline visit to the date of the last visit or attempt to verify subject status. Cause-specific mortality was ascertained to the highest detail possible and then categorized as either death related to (1) IPF, (2) cardiovascular disease, (3) lung cancer (4) other known reasons or (5) unknown.

### Statistical analysis

IBM SPSS Statistics (Version 21.0) was used for the statistical analyses. Missing values were not replaced nor extrapolated. By general convention, p values lower than 0.05 were considered statistically significant. However, no corrections were applied for multiplicity of endpoints and analyses. The number of observations being largely superior to 30, the central limit theorem was invoked and the normality of each variable did not need to be assessed. Parametric statistical tests were used without particular assumptions, each time they were appropriate.

Descriptive statistics were used to characterize the patient population in terms of demographics, survival, comorbidities and pulmonary function.

Median survival times, together with their respective 95% confidence intervals (95% CI), were determined using Kaplan-Meier curves. When different survival curves had to be compared, the log rank test was used to determine if there was a statistically significant difference between groups.

Predictive factors of survival were determined using multivariate Cox proportional hazards regression models and regression models. Adjustments of the model were made on the other co-variables including treatments of the comorbidities. The prognostic impact of comorbidities was determined after generating 9 categories of the GAP-comorbidity co-variable: category 1 = GAP stage I and no comorbidities; category 2 = GAP stage I and 1 to 3 comorbidities; category 3 = GAP stage I and 4 to 7 comorbidities; category 4 = GAP stage II and no comorbidities; category 5 = GAP stage II and 1 to 3 comorbidities; category 6 = GAP stage II and 4 to 7 comorbidities; category 7 = GAP stage III and no comorbidities; category 8 = GAP stage III and 1 to 3 comorbidities; category 9 = GAP stage III and 4 to 7 comorbidities).

Taking into account the results of the adjusted Cox proportional hazards regression model, integrating all comorbidities and co-variables, a “comorbidome” was produced in analogy to [24] and termed as “IPF comorbidome”. It included all comorbidities with more than 10% prevalence in the entire cohort, and comorbidities showing the strongest association with mortality (hazard ratio [HR] >1; 95% confidence interval >1; p<0.05).

## Results

### Patient demographics and baseline characteristics

Patients (N = 272) were 68.5 ± 9.0 years-old (mean ± SD; 44–90 years) at diagnosis. There were 208 males (76.5%) and 64 females (23.5%). 104 were never-smokers (38.2%) and 168 active or former smokers (61.8%). Smoking patients had 20.4 ± 26.1 packs year exposure on average. Fifteen patients (5.5%) had a family history of ILD. Median VC was 73% predicted and DLCO was 44%. Median survival of the entire population was 42 months. All patient characteristics, including pulmonary function, treatments, GAP stages, overall survival, survival duration and reasons for death, are shown in Table 1.

**Table 1:** Baseline characteristics of the patients in terms of pulmonary function, treatments, GAP stages, survival status, survival duration and death reasons.

Parameter	N	n	%	Mean	SD
**Age at first diagnosis (year)**	272	272		68.5	9.0
**Gender**	272				
Male		208	76.5		
Female		64	23.5		
**Smoking status**	272				
Non-smokers		104	38.2		
Active or former smokers		168	61.8		
*Pack years*		168		20.4	26.1
**Family history**	272	15	5.5		
**Pulmonary function**					
VC_max_ (%pred)	272	269		73.4	20.1
FEV1 (%pred)	272	269		80.3	20.7
FEV1/VCmax (%)	272	269		83.7	8.4
DLco (%)	272	264		43.7	19.1
**Treatment of IPF**	272				
Best supportive care (wait and watch)		52	19.1		
Immunosuppressive drugs		134	49.3		
Antioxidants (NAC)		56	20.6		
Anti-fibrotic drugs		30	11.0		
**Comorbidities associated therapies**	272				
Coronary revascularization		36	13.2		
Statins		59	21.7		
Antiplatelet therapy		88	32.4		
Beta-blockers		88	32.4		
ACE inhibitors or angiotensin 1 antagonists		119	43.8		
Other antihypertensive drugs		110	40.4		
Anticoagulants		18	6.6		
*Vitamin K antagonists*		17	6.3		
Antacid drugs		80	29.4		
*Proton pump inhibitors*		76	27.9		
*H2-blockers*		3	1.1		
*Combination proton pump inhibitors and H2-blockers*		1	0.4		
Anti-reflux surgery		0	0.0		
Antidepressants		17	6.3		
Long-acting bronchodilators		41	15.1		
Antidiabetic drugs		41	15.1		
Continuous positive airway pressure		1	0.4		
Pulmonary hypertension drugs		6	2.2		
**GAP stage**	235				
I		89	37.9		
II		103	43.8		
III		43	18.3		
**Survival status**	272				
Alive		101	37.1		
Died		171	62.9		
**Survival duration (month)** Median = 42 ± 4	272	269		58.0	4.2
**Death reason**	171				
Idiopathic pulmonary fibrosis		91	53.2		
Cardiovascular		8	4.7		
Lung cancer		13	7.6		
Other reasons		8	4.7		
Unknown		51	29.8		

SD = Standard Deviation; GAP = Gender, Age and Pulmonary function; %pred = Percentage predicted. N = total number of patients, n = number of patients with available information

HRCT showed a definite UIP pattern in 220 patients (80.9%), possible UIP in 44 patients (16.2%) and unspecified fibrosis in 8 patients (2.9%). Surgical biopsy was not performed in 171 patients (62.9%), and revealed patterns of UIP in 91 patients (33.5%) and probable UIP in 10 patients (3.7%). When combining HRCT and biopsy patterns in accordance with the current ATS/ERS guideline [1], the final diagnosis was definite IPF in 240 (88.2%) and probable IPF in 30 patients (11.8%).

### Comorbidities

The mean number of comorbidities per patient was 2.68 ± 1.83 (0–7). Thirty-one patients (11.4%) had no comorbidities. There were 50 patients (18.4%) with one comorbidity, 56 (20.6%) with 2 comorbidities, 51 (18.8%) with 3 comorbidities, 34 (12.5%) with 4 comorbidities, 28 (10.3%) with 5 comorbidities, 12 (4.4%) with 6 comorbidities and 9 (3.3%) with 7 comorbidities. Overall, 157 patients (57.7%) had 1 to 3 comorbidities and 83 patients (30.5%) had 4 to 7 comorbidities. A total of 212 patients (77.9%) had cardiovascular comorbidity, 21 (7.7%) had central nervous system comorbidity, 133 (48.9%) had pulmonary comorbidity and 60 (22.1%) had cancers. The prevalence of comorbidities in the overall population is shown in S1 Fig.

#### Survival as a function of comorbidities and their treatments

**Fig 1:**
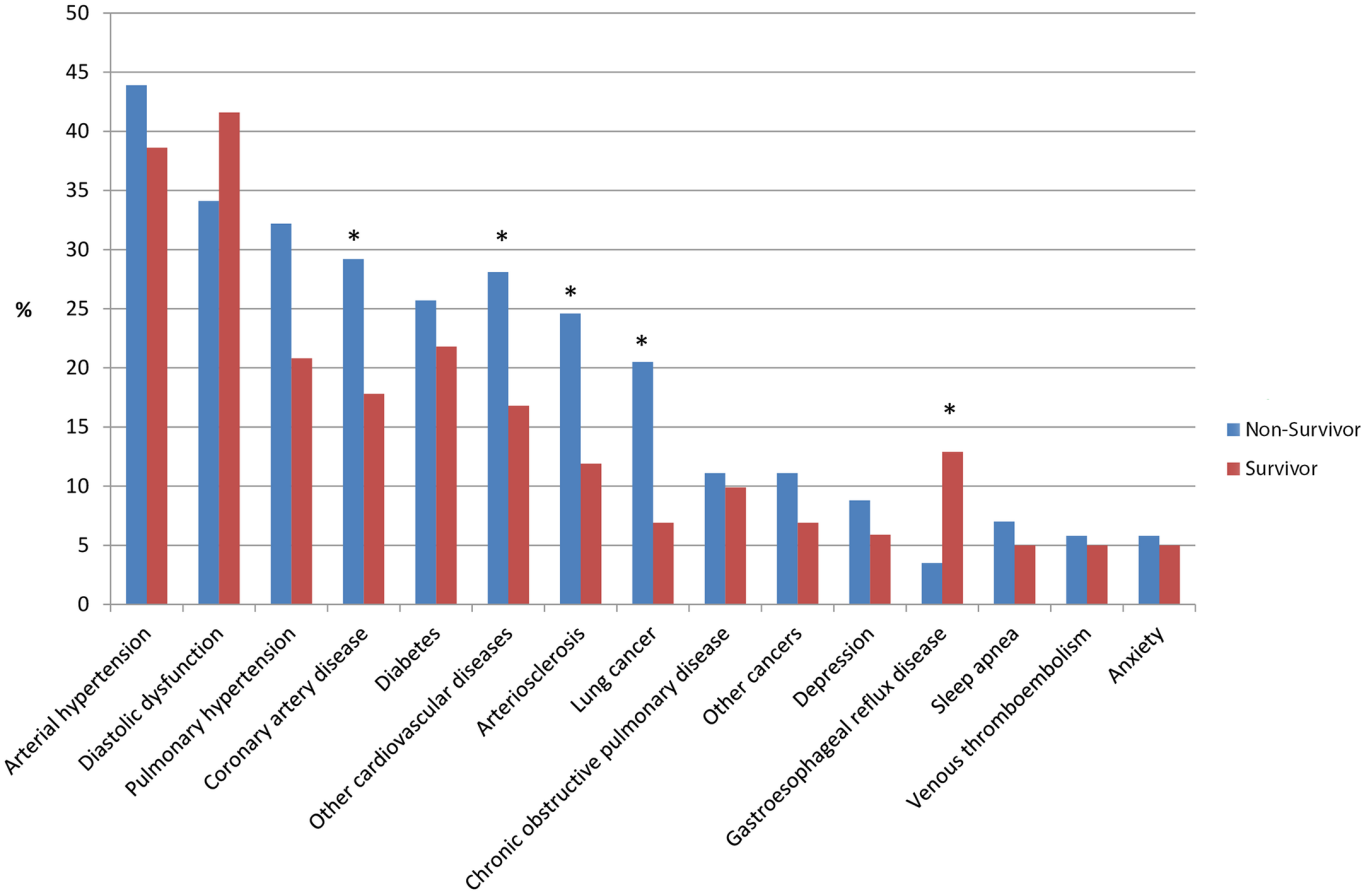
Prevalence of comorbidities in relation to survival status (survivors: red bars, non-survivors: blue bars). The figure also denotes those comorbidities with a significantly (*) different prevalence in non-survivors compared with survivors regardless of their absolute prevalence.

**Fig 2:**
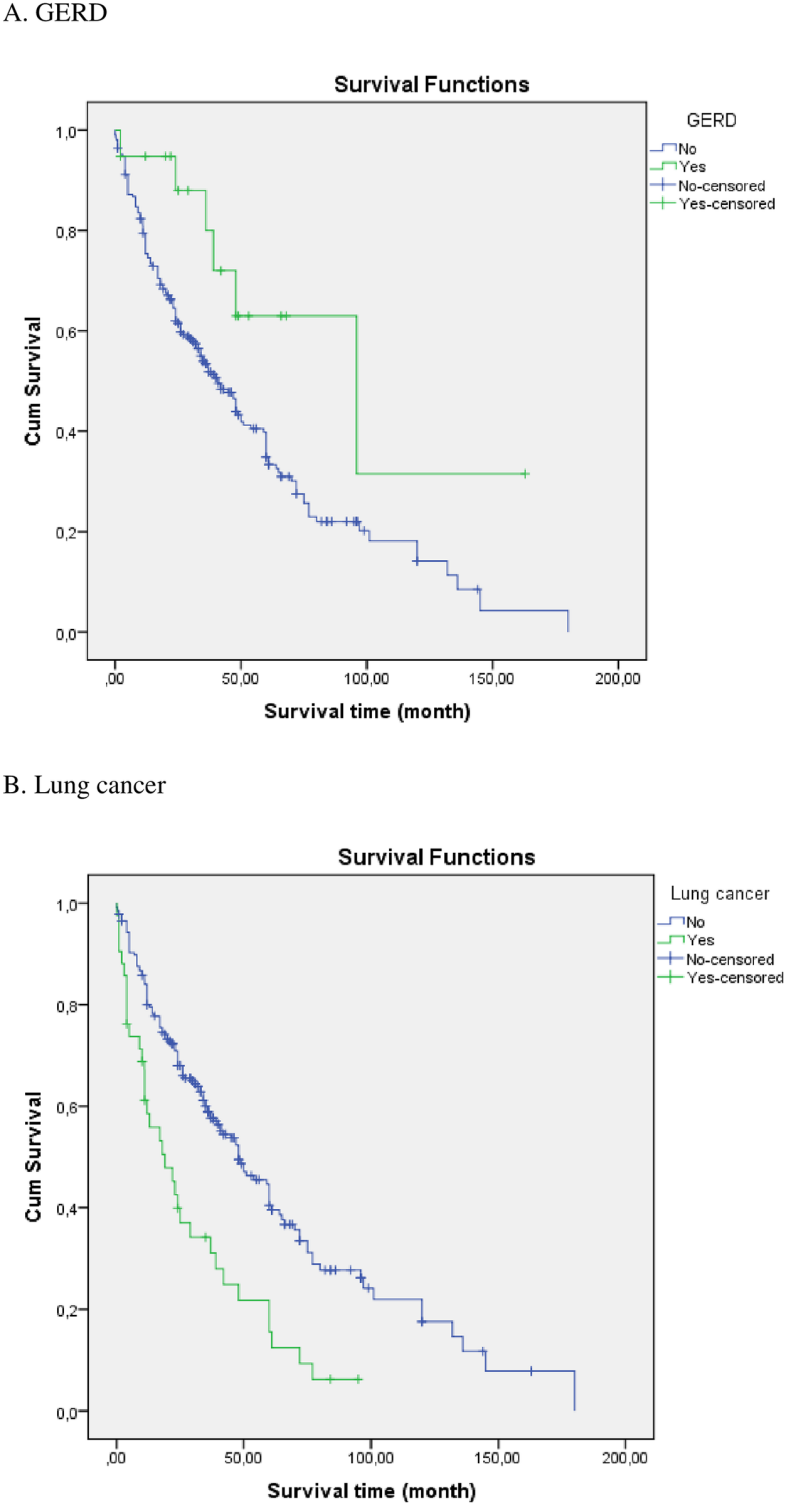
Kaplan-Meier survival curves for patients with (N = 19; median survival 96 months) or without (N = 250; median survival 41 months) (p = 0.026) gastro-oesophageal reflux disease (GERD) and for patients with (N = 42; median survival 19 months) or without (N = 227; median survival 48 months) (p<0.0001) lung cancer.

Prevalence in relation to the survival status and median survivals (with 95% CIs) with regards to the prevalence of specific comorbidities and (organ system clustered) comorbidity categories are shown in Fig 1 and in S1 Table. There was a significant (p<0.05) negative impact of arteriosclerosis, other cardiovascular diseases (mainly valvular heart disease, cardiac arrhythmias, dilated cardiomyopathy), lung cancer, pulmonary and cancer comorbidities on survival. Furthermore, there was a significant (p<0.05) positive impact of gastro-oesophageal reflux disease (GERD) on survival. In a sub-analysis of patients with (n = 19) or without (n = 253) GERD, there were no significant differences between groups in relationship to demographics, smoking habits or functional parameters (data not shown). Kaplan-Meier survival curves for patients with (N = 19) or without (N = 250) GERD and for patients with (N = 42) or without (N = 227) lung cancer are shown in Fig 2.

**Fig 3:**
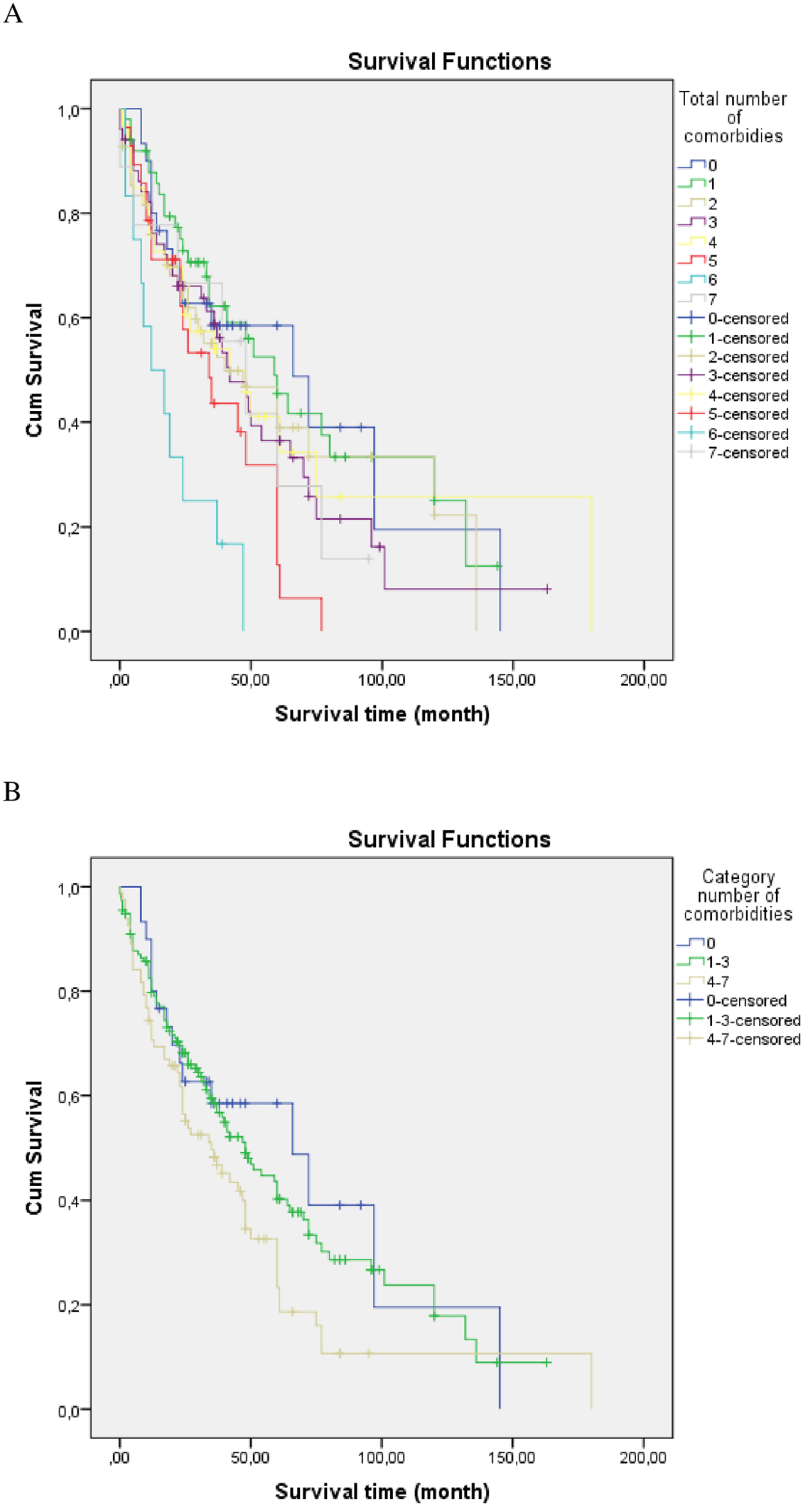
(A) Kaplan-Meier survival curves as a function of the total number of comorbidities. Survival duration decreased as a function of the number of comorbidities (p = 0.004): median survival of 66, 59, 40, 42, 42, 34, 12 months for 0, 1, 2, 3, 4, 5 and 6 comorbidities, respectively. The number of patients with 7 comorbidities was too low (N = 9) to correctly evaluate survival. (B) Kaplan-Meier survival curves as a function of the number of comorbidities clustered in three categories (0, 1–3, 4–7). Survival duration decreased significantly as a function of the number of comorbidities (p = 0.047): 0 comorbidity (N = 30; median survival 66 months), 1–3 comorbidities (N = 156; median survival 48 months) and 4–7 comorbidities (N = 82; median survival 35 months).

A statistically significant difference in terms of survival time was also found as a function of the total number of comorbidities (p = 0.004). Median survival decreased from 66 months when no comorbidities were reported to 12 months when 6 comorbidities were reported. Also, a significant difference in terms of survival time as a function of the number of comorbidities regrouped in three categories was detected (p = 0.047). Median survival declined from 66 months when no comorbidities were reported to 48 months when 1 to 3 comorbidities were reported and 35 months when 4 to 7 comorbidities were prevalent (Fig 3).

In a multivariate Cox proportional hazards regression model the GAP-comorbidity co-variable was not a significant predictor of death (p>0.05). However, within each GAP stage, there was a tendency for an increase in the odds ratio of death as a function of the number of comorbidities. Within GAP stage II, the odds ratios were 0.980, 1.714 and 1.780 for 0, 1–3 and 4–7 comorbidities respectively. The odds ratio of death was multiplied by 6 when going from GAP stage I with 1–3 comorbidities (0.486) to GAP stage III with 4–7 comorbidities (3.000).

In a further analysis, the impact of treatments of comorbidities was assessed. The following therapies were significantly more frequent in deceased versus alive patients: statins (25.7% versus 15%, p = 0.047), antiplatelet drugs (40% versus 19.8%, p = 0.001), beta-blockers (37.4% versus 23.8%, p = 0.023), anticoagulants (9.4% versus 2%, p = 0.021), and antidiabetic drugs (18.8% versus 8.9%, p = 0.035). The following drugs did not differ significantly: ACE inhibitors or angiotensin 1 antagonists (48.0% versus 36.6%, p = 0.077), other antihypertensive drugs (43.3% versus 35.6%, p = 0.250), antidepressants (7% versus 5%, p = 0.609), coronary revascularisation (15.8% versus 8.9%, p = 0.138), long actin bronchodilators (17% versus 11.9%, p = 0.296), antacid drugs (29.2% versus 29.7%, p = 1.000), continuous positive airway pressure (0% versus 1%, p = 0.373) and drugs for the treatment of pulmonary hypertension drugs (1.8% versus 3.0%, p = 0.674).

**Fig 4:**
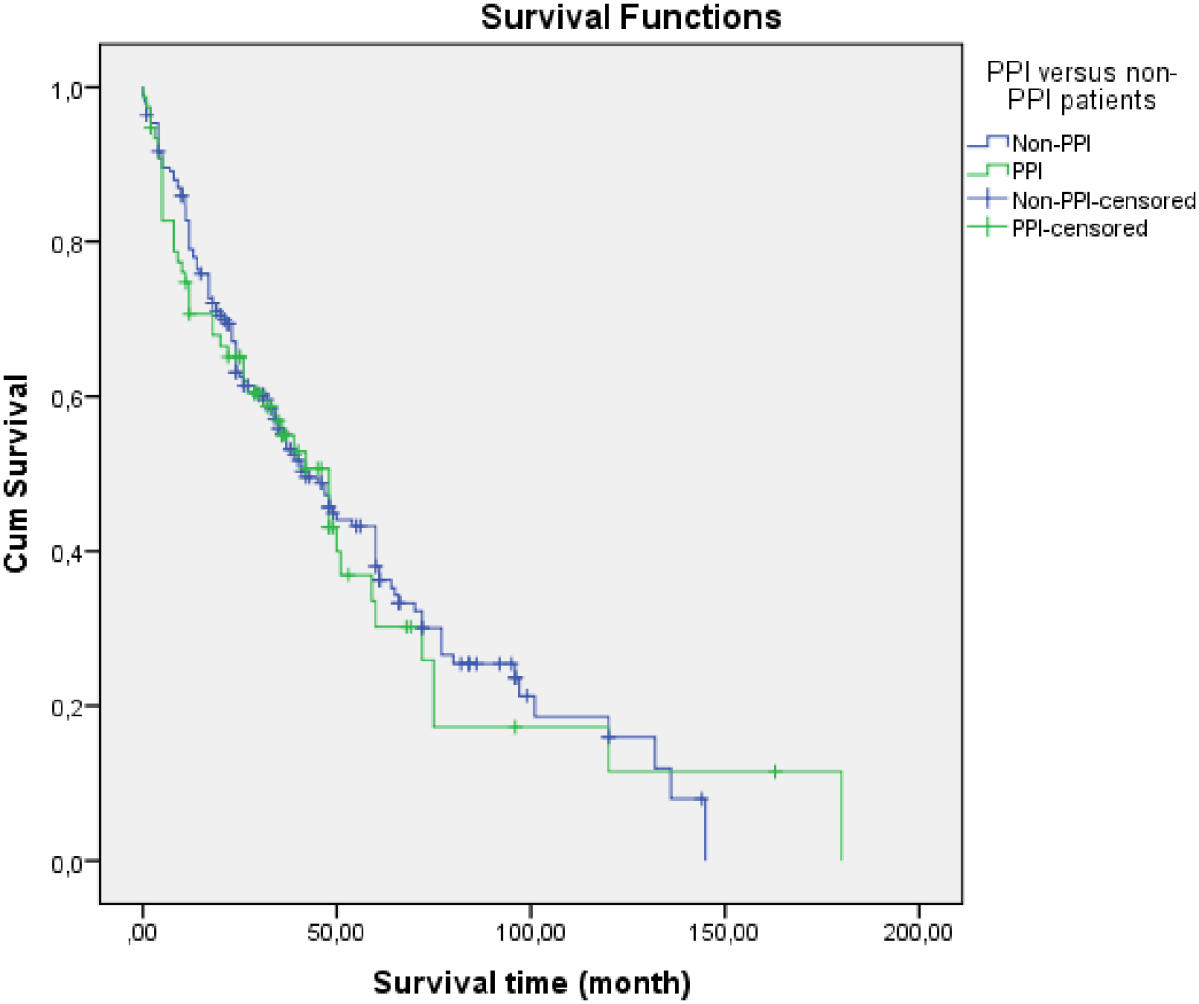
Kaplan-Meier survival curves for patients receiving proton pump inhibitors (PPI) at baseline (n = 76, median survival 48 months) versus patients (n = 193, median survival 42 months) without PPI at baseline (p = 0.718).

The comparison of patients receiving proton pump inhibitors (PPI) at baseline (n = 76) versus patients (n = 193) without PPI at baseline (excluding those with H2-blockers, n = 3) did not show differences in median survivals with 48 months in patients with PPI versus 42 months in patients without-PPI at baseline (p = 0.718). Kaplan-Meier survival curves for patients with or without (N = 250) PPI at baseline are shown in Fig 4.

With regards to patients taking anticoagulants (n = 18), mainly Vitamin K antagonists, there was a difference in survival. Patients with anticoagulants at baseline had a median survival of 42 months compared to patients without anticoagulants (n = 254) at baseline with 31 months. However, this did not reach statistical significance (p = 0.235).

In an analysis of patients with at least one cardiovascular comorbidity, a predictive model of survival using multivariate Cox proportional hazards regression including all types of cardiovascular treatments did not show a statistically significant influence on survival (data not shown).

A predictive model of survival, adjusted on the basis of all the other observed or measured variables, was calculated using multivariate Cox proportional hazards regression. Statistically significant (p<0.05) variables were gender (female more prone to death), diastolic dysfunction (favourable to survival), arteriosclerosis (favourable to death), other cardiac diseases (favourable to death), GERD (favourable to survival), lung cancer (favourable to death), VC max (higher values favourable to survival), FEV1 (higher values favourable to death), lung biopsy UIP (favourable to death) and treatment with anti-fibrotics (favourable to survival) and treatment with antacids (favourable to death). Significant (p<0.01) predictors were a high VC max at baseline (p = 0.003; HR 0.918), diastolic dysfunction (p = 0.002; HR = 0.473) and GERD (p = 0.007; HR = 0.270) as favourable to survival. Favourable to death were treatment with antacids (p = 0.004; HR 2.152) other cardiac diseases (p = 0.005; HR = 1.943) and lung cancer (p<0.0001; HR = 2.924) (Table 2).

**Table 2:** Predictive model of survival, adjusted on the basis of all the other observed or measured variables (all entered at the same time), calculated using multivariate Cox proportional hazards regression.

	P value	Hazard ratio (HR)	95.0% CI around HR	
			Lower	Upper
**Age at diagnosis**	0.316	1.017	0.984	1.052
**Gender**	0.023	2.318	1.121	4.791
**Coronary artery disease**	0.383	1.342	0.693	2.601
**Diastolic dysfunction**	0.002	0.473	0.294	0.763
**Diabetes**	0.227	0.646	0.318	1.314
**Sleep apnea**	0.182	0.499	0.180	1.385
**Pulmonary hypertension (PH)**	0.734	1.086	0.676	1.744
**COPD**	0.236	1.592	0.737	3.438
**Arterial hypertension**	0.697	1.097	0.690	1.744
**Arteriosclerosis**	0.039	1.694	1.027	2.794
**VTE**	0.291	1.581	0.676	3.694
**Other cardiac diseases**	0.005	1.943	1.226	3.078
**GERD**	0.007	0.270	0.104	0.702
**Depression**	0.232	2.101	0.622	7.097
**Anxiety**	0.896	0.906	0.207	3.973
**Lung cancer**	<0.0001	2.924	1.620	5.278
**Other cancers**	0.702	1.146	0.569	2.311
**Smoking**	0.859	1.058	0.568	1.971
**Number of packs per year**	0.371	1.006	0.992	1.020
**Family history for ILD**	0.304	0.639	0.272	1.501
**VCmax (%) at baseline**	0.003	0.918	0.869	0.970
**FEV1 (%) at baseline**	0.016	1.065	1.012	1.120
**TiFF (%) at baseline**	0.356	0.978	0.933	1.025
**DLCO (%) at baseline**	0.223	0.992	0.980	1.005
**Immunosuppressive drugs**	0.949	1.019	0.571	1.819
**Antioxidants (NAC)**	0.399	1.328	0.687	2.569
**Anti-fibrotic drugs**	0.025	0.260	0.080	0.843
**Coronary revascularisation**	0.305	0.657	0.295	1.466
**Statins**	0.969	1.012	0.547	1.875
**Antiplatelet therapy**	0.158	1.464	0.862	2.488
**Beta-blockers**	0.592	1.158	0.678	1.978
**ACE inhibitors or Angiotensin 1 antagonists**	0.138	1.468	0.884	2.439
**Other antihypertensive drugs**	0.055	0.633	0.396	1.011
**Anticoagulants**	0.669	0.834	0.363	1.915
**Antacid drugs**	0.004	2.152	1.277	3.629
**Antidepressants**	0.727	0.839	0.314	2.246
**Long actin bronchodilators**	0.149	0.657	0.371	1.162
**Antidiabetic drugs**	0.125	1.831	0.845	3.970
**Pulmonary hypertension drugs**	0.334	2.392	0.408	14.018

COPD = Chronic Obstructive Pulmonary Disease; GERD = Gastro-Esophageal Reflux Disease; VTE = Venous Thrombo-Embolism; %pred = Percentage predicted

**Fig 5:**
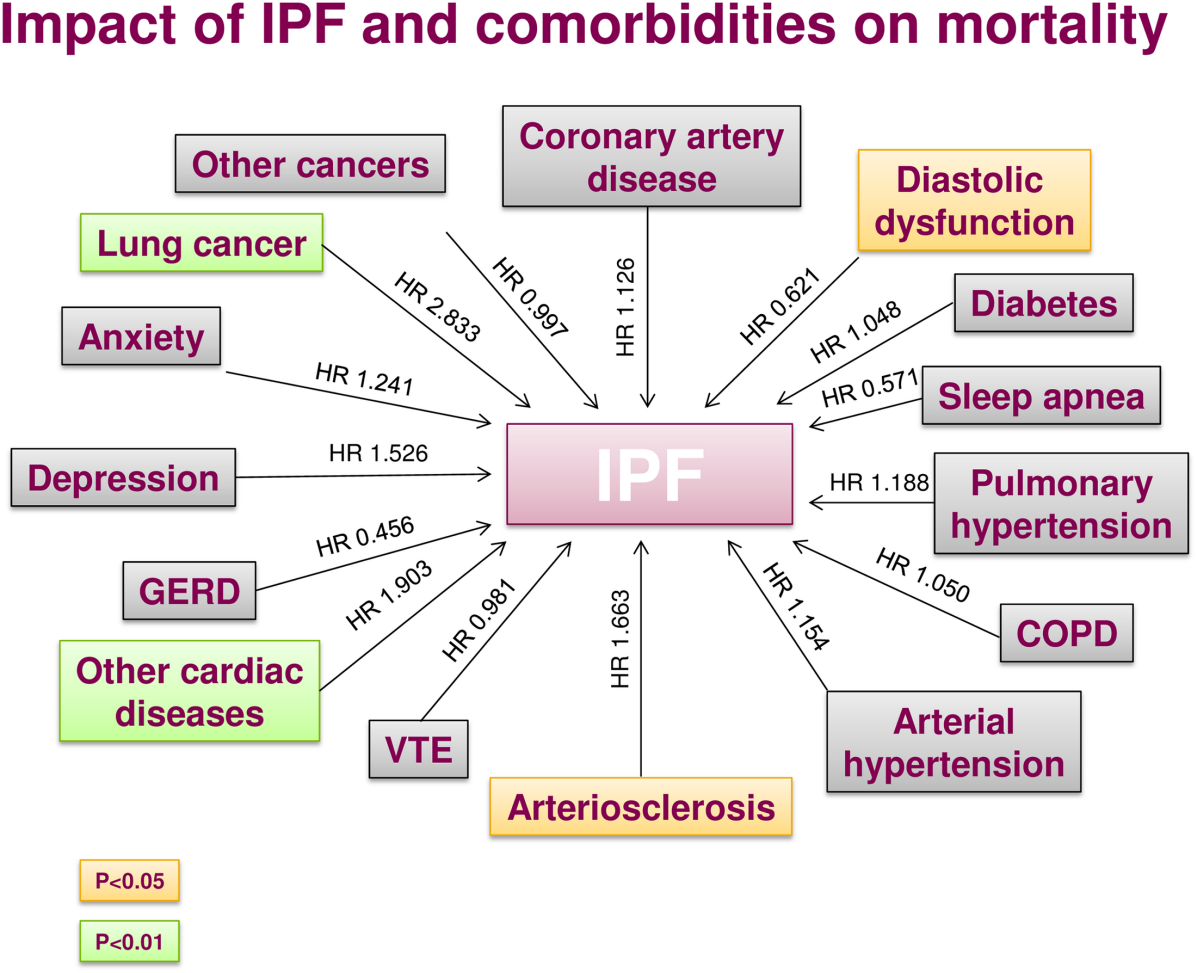
Impact of idiopathic pulmonary fibrosis and comorbidities on mortality. Hazard ratios (HR) have been determined using a predictive multivariate Cox proportional hazards regression model.

Fig 5 represents the impact of comorbidities on IPF and survival.

#### Comorbidome

**Fig 6:**
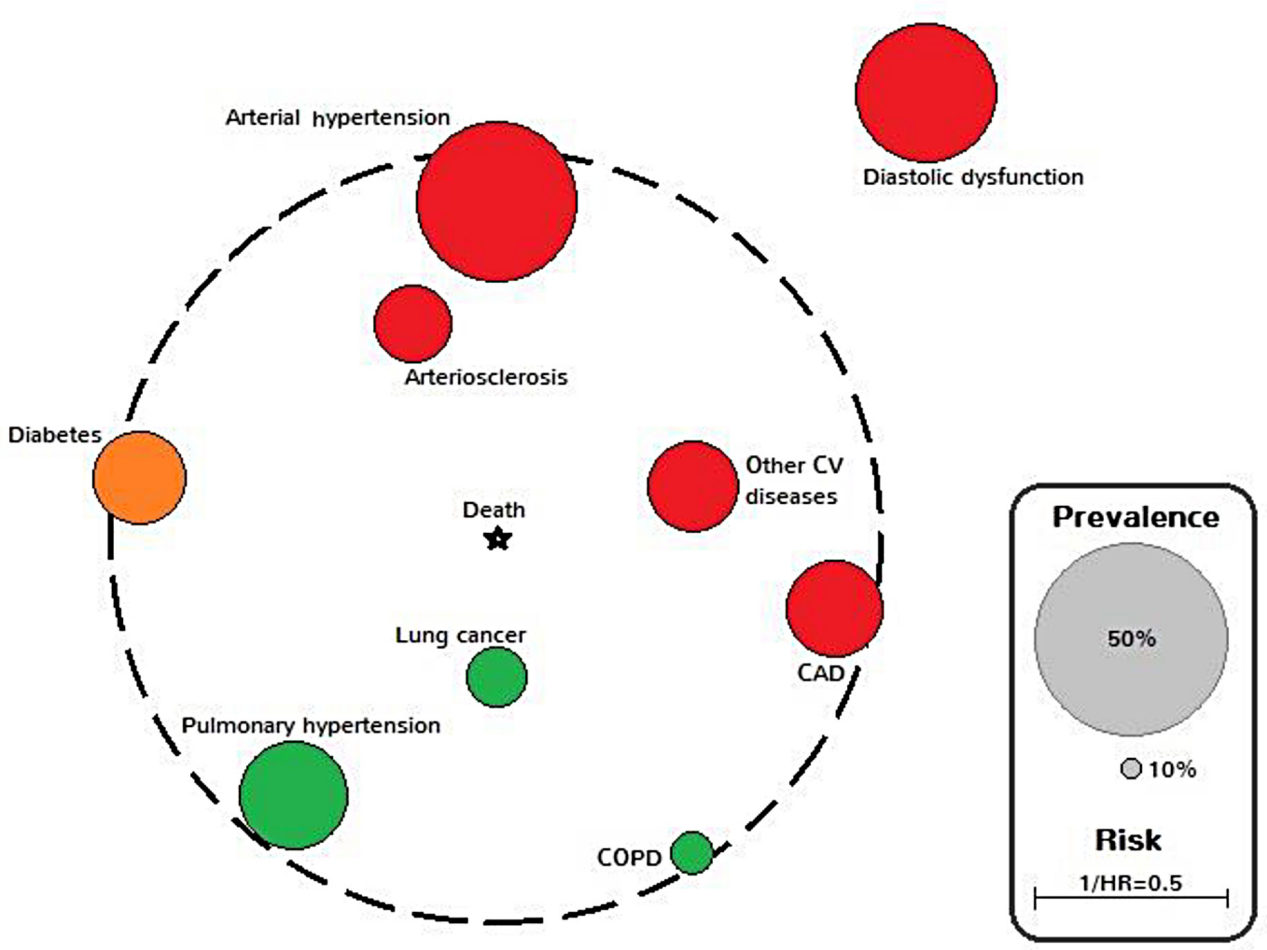
Graphic expression (comorbidome) of comorbidities with more than 10% prevalence in the entire cohort, and those comorbidities with the strongest association with mortality (hazard ratio [HR] >1; 95% confidence interval >1; p<0.05). The area of the circle relates to the prevalence of the disease. The proximity to the centre (mortality) expresses the strength of the association between the disease and risk of death. This was scaled from the inverse of the HR (1/HR). All bubbles associated with a statistically significant increase in mortality are fully inside the dotted orbit (1/HR <1). Bubble colours represent organ systems or disease clusters (cardiovascular = red, pulmonary = green, others = orange).

Nine comorbidities had an overall prevalence higher than 10%. Their prevalence and the strength of their association with mortality are presented in Fig 6 as an orbital bubble chart in analogy to Divo et al. [24] and was termed “IPF comorbidome”. Mortality is fixed at the centre and comorbidities are represented as bubbles or “planets” with their diameters proportional to their prevalence. Each “planet” is positioned in a radial “orbit” with the distance to the centre scaled from the inverse of the hazard ratio (1/HR). The closer the comorbidity is to the centre, the higher the conferred risk. All bubbles associated with a statistically significant increase in mortality are fully inside the dotted orbit (1/HR <1).

## Discussion

We here report about an observational retrospective long-term analysis of IPF patients who were screened for comorbidities. Our study has several new important findings. (1) The overall number of comorbidities is significantly associated with survival in IPF. (2) Several comorbidities have a significant influence on survival in IPF; some had a favourable effect, such as GERD, while others had a detrimental effect, such as lung cancer which has the most unfavourable association in IPF (3). In an “IPF comorbidome” which we developed in analogy to the COPD comorbidome described by Divo et al. [24] we were able to establish a new relationship of IPF and comorbidities. This novel finding describes the spatial expression of the prevalence of comorbidities and their strength of association with mortality in IPF. Our “IPF comorbidome” visually conveys the prevalence of concomitant diseases and the risk of death for an individual IPF patient. We propose that the “IPF comorbidome” might be useful as a predictor for the risk of death in IPF.

In the light of new therapeutic options in IPF [25, 26] it becomes even more important to carefully document and understand critical comorbidities as they are not only common but also have a significant influence on quality of life, functional impairment and survival. Whilst often described as being a substantial clinical challenge, there is a paucity of clinical studies and the real impact of comorbidities on survival in IPF is not well characterised. Our study contributes to the literature by providing comprehensive data on the frequency of comorbidities in a typical IPF cohort also in correlation to their GAP status and in relation to the underlying therapy. A recent study from Denmark reported by Hyldgaard and co-workers described 121 patients diagnosed with IPF between 2003 and 2009 [27]. In this study, the most frequently observed comorbidities were cardiovascular diseases, depression, arterial hypertension and diabetes mellitus with incidences comparable to our cohort. In contrast, the incidence for lung cancer was considerably lower than in our cohort. Differences might be explained by the longer study period in our cohort and the larger number of patients. Further, incidences of distinct comorbidities could be influenced by local differences, like ethnicity, diet habits, smoking status or physical status as BMI. For some important concomitant diseases, the prevalence was similar, e.g. for GERD. The INSIGHTS-IPF registry currently follows one of the largest IPF cohorts in Europe, with more than 500 patients from 19 centres in a prospective observation since 2012 [3]. The most frequently observed comorbidities in this analysis were arterial hypertension, coronary heart disease and diabetes mellitus—again comparable to our study. Surprisingly, only 1% of INSIGHTS-IPF participants had lung cancer whereas the prevalence of lung cancer in our study was much higher (15%). This might be explained by the prospective nature of the INSIGHTS-IPF registry and a potential inclusion bias, that deters investigators from including patients with IPF and concomitant lung cancer in registries like INSIGHTS-IPF as they maybe more likely to be lost to follow up.

The influence of lung cancer on survival in IPF is explicitly shown in our comorbidome approach and the analyses of reasons for death. Previous studies have revealed a high incidence of lung cancer in IPF. Yet, the true cumulative incidence of lung cancer after the diagnosis of IPF and its predictive factors at the initial diagnosis of IPF remain unknown. Ozawa and colleagues found in their study of 103 IPF patients that 20.4% developed lung cancer during an observation period of up to 10 years with increasing cumulative incidence with the duration of follow up [28]. Tomassetti and co-workers found that lung cancer in IPF patients has a significant negative impact on survival. They determined that the difference in mortality seen in their study was not due to worsening of pulmonary fibrosis, but mainly to lung cancer progression and complications of its treatment [11], very much comparable to analyses reported before by our group [10].

Gastroesophageal reflux (GER) is an often reported condition in the IPF population. Depending on the study and type of assessment, the presence of typical reflux symptoms and the diagnosis of GERD range from 0–94% [27, 29, 30]. Yet, the prognostic impact of a GERD diagnosis on IPF and its pathophysiologic interaction is still unclear and the impact of antacid therapy on outcomes in IPF currently under debate [21, 31, 32]. Surprisingly, in our study we found a beneficial effect of GERD on survival. This is in line with a retrospective study also reporting that the median survival time for IPF patients with GERD is significantly longer than in IPF patients without GERD [15]. Potential causes for this observation are still unclear but may be related to a lead time bias effect, i.e. an earlier diagnosis due to GERD-related pulmonary symptoms thereby a longer survival compared to later diagnosed patients. Another -currently discussed- argument is the potential impact of antacid therapy, mainly proton pump inhibitors on the course of IPF. A retrospective analysis reported that antacid therapy is associated with longer survival in IPF patients [15] and a combined posthoc analysis of the IPFnet trials also described that IPF patients taking antacid treatment had a smaller decrease in FVC [16]. As a reason for this observation the authors speculated that antacid treatment might decrease the frequency of acute exacerbations, potentially by reducing the acidity of the microaspirate [15, 16]. On the basis of these results the current IPF treatment guideline gives a conditional recommendation for the use of proton pump inhibitors in patients with IPF, although with very low confidence in estimates of effect [21]. However, recently published post-hoc analyses from the placebo groups of 3 trials of pirfenidone (CAPACITY and ASCEND) do not support this previously reported relationship between antacid therapy and reduced IPF progression [32]. Also in the analyses reported here, there was no significant impact of PPI on survival. Nevertheless, the true pathogenetic and prognostic role of GERD in IPF is still unclear and prospective data on the impact of antacids on IPF are required.

Interestingly, some potential comorbidities in IPF are not captured in any of the reported studies, including ours. Little information is known about concomitant liver diseases, chronic renal failure and degenerative joint disease. This might be due to underreporting or due to true absence of disease associations. Future registry studies will need to address this issue.

It is intriguing to ask whether there is more than a simple association between certain comorbidities and IPF. Shared risk factors such as cigarette smoking, which is common for patients with IPF, lung cancer and CAD support the assumption that some are primarily linked by risk factors. Another association might be the process of aging which is related to telomerases. Telomerase mutations in TERT or TERC which cause telomere shortening increase the susceptibility to IPF [33] and there is also considerable evidence that telomere attrition is associated with, and possibly causative of cancer [34] as well as of emphysema [35]. This makes the hypothesis that IPF and some of its comorbidities are a “combined” disease of aging reasonable. With regards to lung cancer also other common pathogenetic pathways with IPF may exist [36], while diabetes may influence the progression or initiation of IPF by hyperglycaemia associated pulmonary inflammation [37, 38]. With regards to gastroesophageal reflux disease (GERD), repetitive microaspiration could lead to chronic inflammation causing fibrotic proliferation [39–41].

Treatment of comorbidities may have the potential to improve quality of life in IPF patients. Until we have more robust prospective data, one may consider that comorbid conditions should be treated the same way as in non-IPF patients. Whether the treatment of comorbidities impacts survival in IPF has still to be established. While theoretically treatment of underlying comorbidities may have a positive impact, recently it was reported that anticoagulation for medical indication may be associated with more detrimental effects [42]. However, our data did not reveal a significant impact of comorbidities associated therapies which are yet limited by its retrospective nature and that only baseline conditions were assessed. Some possible treatments of comorbidities in IPF include life style modification and weight reduction in case of obesity, treatment of symptomatic GERD and sleep apnoea, reducing cardiovascular risk, smoking cessation and long term oxygen therapy, which has in addition a positive influence on PH. Also pulmonary rehabilitation [43, 44] can positively impact depression and cardiovascular diseases.

The present study has several limitations. First, it was a retrospective, single centre study. Although comorbidities were actively screened also with the help of a structured questionnaire [22], they were physician and self-reported, which could result in some underreporting. This could explain why we found lower incidences for some comorbidities than reported before, e.g. for GERD [27]. Also, the influence of IPF treatment and of specific comorbidities on the course of IPF is unknown and the study results could have been influenced by the fact that therapeutic recommendations for IPF treatment changed significantly during the study period [1, 21, 45]. Furthermore, as the data in this study come from patients of a tertiary referral centre for ILD, the patient characteristics and comorbidities might differ from those of other cohorts and might inherit a bias towards more sick patients. Finally, some results could have changed if comorbidities and their treatments were assessed longitudinally.

In summary, we describe a significant and clinically meaningful association of comorbidities with an elevated risk of death in patients with IPF. In this context, the “IPF comorbidome” proposed here may support the concept of personalised medicine in IPF. Future research should be undertaken to analyse the influence of treatment of comorbidities on IPF outcome and IPF related factors such as quality of life and symptoms.

## Supporting Information

S1 Fig Prevalence of the comorbidities in the total study population. CV = Cardiovascular; COPD = Chronic Obstructive Pulmonary Disease; GERD = Gastro-Esophageal Reflux Disease; VTE = Venous Thrombo-Embolism.
(TIF)

S1 Table Median survival duration (with 95% CIs) in the presence or absence of individual comorbidities (classified according to their prevalence) or comorbidity categories clustered by body systems. CV = Cardiovascular; COPD = Chronic Obstructive Pulmonary Disease; GERD = Gastro-Esophageal Reflux Disease; VTE = Venous Thrombo-Embolism. Cardiovascular morbidity included coronary artery disease, diastolic dysfunction, hypertension, arteriosclerosis, VTE and other cardiovascular diseases. Central nervous system comorbidity included depression and anxiety. Pulmonary comorbidity included sleep apnea, pulmonary hypertension, COPD and lung cancer. Cancer comorbidity included lung cancer and other cancers.
(DOCX)
