# Supplementary figures and images for: Impact of Comorbidities on Mortality in Patients with Idiopathic Pulmonary Fibrosis
Source: PLoS One. 2016 Mar 29;11(3):e0151425. doi: 10.1371/journal.pone.0151425 (PMC4811578; doi:10.1371/journal.pone.0151425)

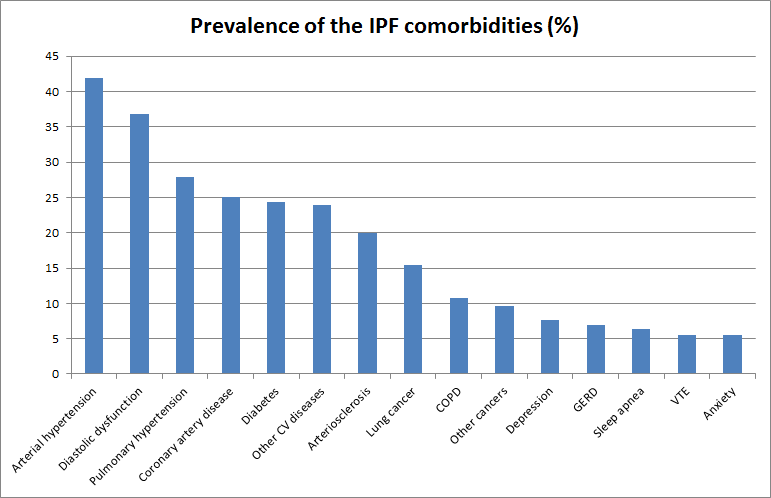

Supplement: S1 Fig — CV = Cardiovascular; COPD = Chronic Obstructive Pulmonary Disease; GERD = Gastro-Esophageal Reflux Disease; VTE = Venous Thrombo-Embolism. (TIF) [file pone.0151425.s001.tif]
