# Supplementary material for: Impact of Comorbidities on Mortality in Patients with Idiopathic Pulmonary Fibrosis
Source: PLoS One. 2016 Mar 29;11(3):e0151425. doi: 10.1371/journal.pone.0151425 (PMC4811578; doi:10.1371/journal.pone.0151425)
Supplement: S1 Table — CV = Cardiovascular; COPD = Chronic Obstructive Pulmonary Disease; GERD = Gastro-Esophageal Reflux Disease; VTE = Venous Thrombo-Embolism. Cardiovascular morbidity included coronary artery disease, diastolic dysfunction, hypertension, arteriosclerosis, VTE and other cardiovascular diseases. Central nervous system comorbidity included depression and anxiety. Pulmonary comorbidity included sleep apnea, pulmonary hypertension, COPD and lung cancer. Cancer comorbidity included lung cancer and other cancers. (DOCX) [file pone.0151425.s002.docx]

Table 1 suppl. Median survival duration (with 95% CIs) in the presence or absence of individual comorbidities (classified according to their prevalence) or comorbidity categories clustered by body systems

| **Comorbidity or comorbidity category** | **Presence or**  **Absence** | **Median (month)** | **95% confidence interval** | | **Log rank**  **test p-value** |
| --- | --- | --- | --- | --- | --- |
|  |  |  | **Lower limit** | **Upper limit** |  |
| Arterial hypertension | Presence | 42 | 33.5 | 50.5 | 0.115 |
|  | Absence | 48 | 28.5 | 67.5 |  |
| Diastolic dysfunction | Presence | 48 | 41.9 | 54.0 | 0.150 |
|  | Absence | 37 | 28.0 | 46.0 |  |
| Pulmonary hypertension | Presence | 32 | 12.6 | 51.4 | 0.221 |
|  | Absence | 48 | 33.1 | 62.9 |  |
| Coronary artery disease | Presence | 37 | 17.1 | 56.9 | 0.160 |
|  | Absence | 47 | 37.0 | 57.0 |  |
| Diabetes | Presence | 37 | 28.0 | 46.0 | 0.199 |
|  | Absence | 48 | 38.0 | 58.0 |  |
| Other CV diseases | Presence | 26 | 10.7 | 41.3 | 0.016 |
|  | Absence | 48 | 34.7 | 61.3 |  |
| Arteriosclerosis | Presence | 31 | 15.0 | 47.0 | 0.002 |
|  | Absence | 48 | 38.1 | 57.9 |  |
| Lung cancer | Presence | 19 | 7.3 | 30.7 | <0.0001 |
|  | Absence | 48 | 35.6 | 60.4 |  |
| COPD | Presence | 37 | 0.0 | 80.4 | 0.900 |
|  | Absence | 45 | 37.5 | 52.5 |  |
| Other cancers | Presence | 37 | 19.5 | 54.5 | 0.524 |
|  | Absence | 47 | 38.7 | 55.3 |  |
| Depression | Presence | 35 | 17.2 | 52.8 | 0.104 |
|  | Absence | 47 | 38.3 | 55.7 |  |
| GERD | Presence | 96 | 26.5 | 165.5 | 0.026 |
|  | Absence | 41 | 33.1 | 48.9 |  |
| Sleep apnea | Presence | 42 | 25.5 | 58.5 | 0.860 |
|  | Absence | 45 | 36.4 | 53.6 |  |
| VTE | Presence | 50 | 28.8 | 71.2 | 0.828 |
|  | Absence | 42 | 34.4 | 49.6 |  |
| Anxiety | Presence | 34 | 17.3 | 50.7 | 0.240 |
|  | Absence | 45 | 37.2 | 52.8 |  |
| Cardiovascular system | Presence | 45 | 37.5 | 52.5 | 0.460 |
|  | Absence | 41 | 0.0 | 83.5 |  |
| Central nervous system | Presence | 35 | 17.2 | 52.8 | 0.104 |
|  | Absence | 47 | 38.3 | 55.7 |  |
| Pulmonary system with lung cancer | Presence | 33 | 20.8 | 45.2 | 0.005 |
|  | Absence | 54 | 39.8 | 68.2 |  |
| Pulmonary system without lung cancer | Presence | 37 | 25.5 | 48.5 | 0.217 |
|  | Absence | 50 | 34.7 | 65.3 |  |
| Cancer | Presence | 24 | 20.1 | 27.9 | <0.0001 |
|  | Absence | 48 | 36.0 | 60.0 |  |

CV = Cardiovascular; COPD = Chronic Obstructive Pulmonary Disease; GERD = Gastro-Esophageal Reflux Disease; VTE = Venous Thrombo-Embolism. Cardiovascular morbidity included coronary artery disease, diastolic dysfunction, hypertension, arteriosclerosis, VTE and other cardiovascular diseases. Central nervous system comorbidity included depression and anxiety. Pulmonary comorbidity included sleep apnea, pulmonary hypertension, COPD and lung cancer. Cancer comorbidity included lung cancer and other cancers.
